# Self-Compassion Interventions to Target Secondary Traumatic Stress in Healthcare Workers: A Systematic Review

**DOI:** 10.3390/ijerph20126109

**Published:** 2023-06-12

**Authors:** Annabel Rushforth, Mia Durk, Gabby A. A. Rothwell-Blake, Ann Kirkman, Fiona Ng, Yasuhiro Kotera

**Affiliations:** 1 College of Health, Psychology and Social Care, University of Derby, Derby DE22 1GB, UK; a.kirkman2@derby.ac.uk; 2 Institute of Psychology, Psychiatry and Neuroscience, King’s College London, London WC2R 2LS, UK; m.durk98@gmail.com; 3 Independent Researcher, Sheffield S1 4RG, UK; gabbyrothwellblake7@gmail.com; 4 School of Health Sciences, University of Nottingham, Nottingham NG7 2TU, UK; fiona.ng@nottingham.ac.uk (F.N.); yasuhiro.kotera@nottingham.ac.uk (Y.K.)

**Keywords:** secondary traumatic stress, self-compassion, compassion fatigue, health care worker, systematic review

## Abstract

Healthcare professionals’ wellbeing can be adversely affected by the intense demands of, and the secondary traumatic stress associated with, their job. Self-compassion is associated with positive wellbeing outcomes across a variety of workforce populations and is potentially an important skill for healthcare workers, as it offers a way of meeting one’s own distress with kindness and understanding. This systematic review aimed to synthesise and evaluate the utility of self-compassion interventions in reducing secondary traumatic stress in a healthcare worker population. Eligible articles were identified from research databases, including ProQuest, PsycINFO, ScienceDirect, Google Scholar, and EBSCO. The quality of non-randomised and randomised trials was assessed using the Newcastle–Ottawa Scale. The literature search yielded 234 titles, from which 6 studies met the inclusion criteria. Four studies reported promising effects of self-compassion training for secondary traumatic stress in a healthcare population, although these did not use controls. The methodological quality of these studies was medium. This highlights a research gap in this area. Three of these four studies recruited workers from Western countries and one recruited from a non-Western country. The Professional Quality of Life Scale was used to evaluate secondary traumatic stress in all studies. The findings show preliminary evidence that self-compassion training may improve secondary traumatic stress in healthcare professional populations; however, there is a need for greater methodological quality in this field and controlled trials. The findings also show that the majority of research was conducted in Western countries. Future research should focus on a broader range of geographical locations to include non-Western countries.

## 1. Introduction

Secondary traumatic stress (STS) has been defined as “the natural, consequent behaviours and emotions resulting from knowledge about a traumatising event experienced by a significant other” [1]. Indirect exposure to trauma, combined with the affective reaction of empathy provision, may lead to empathy-based strain and STS [2]. Ludick and Figley [3] proposed the multidimensional compassion fatigue resilience (CFR) model as the current mechanism of STS onset. The model comprises three sectors: empathic stance, STS, and CFR. An innate level of compassion and drive towards helping others likely influences individual vulnerability towards STS. An accumulation of empathic stress and negative affect while lacking proper intervention may well lead to STS [4,5]. CFR demonstrates adaptive coping and the potential prevention of STS through promoting the prioritisation of personal wellbeing whilst simultaneously being able to presently appear in support of others.

Individuals who professionally support those who are suffering or traumatised may be exposed to indirect trauma due to the intake of patients’ traumatic stories [6]. Therefore, healthcare workers (HCWs) can frequently be exposed to secondary trauma. The concept of STS has been recognsied by different professionals working with trauma victims in mental health [7], where professionals providing first-line support to traumatised patients were suggested as being at the greatest risk of STS. However, STS is not restricted to mental healthcare professionals. Beck [8] detailed how STS is understood as likely among HCWs. Professionals, such as paramedics, nurses, and doctors, who directly contact patients and may witness traumatic injuries are at risk of STS. The recent COVID-19 pandemic has further highlighted the debilitating impacts of secondary traumatisation and STS on HCWs and the need for novel interventions [9].

Studies conducted among nurses working in emergency departments revealed the prevalence of STS within this population. A study in Scotland detailed that 75% of nurses presented with at least one STS symptom [10]; studies conducted in Ireland and Jordan demonstrated that 64% of nurses met the criteria for STS and 52.3% presented with high or severe levels of STS, respectively [11,12]. Among emergency doctors in the USA, studies demonstrated that almost 13% met the criteria for STS and nearly 34% presented with at least one STS symptom [13]. A systematic review included a study that demonstrated that 54% of doctors met the criteria for STS; however, the STS intensity among doctors within the other studies presented was low [14]. Finally, prior to the COVID-19 pandemic, STS among HCWs was described to range from 4% to 13% [15]. Orrù et al. [9] revealed that during the pandemic, STS symptoms exceeded 40% across HCWs; prevalence was greater for those on the frontline (47.5%) and in HCWs who witnessed patients’ deaths (67.1%).

Self-compassion refers to how we understand and act kindly towards ourselves when faced with personal failure, suffering, and feelings of inadequacy [16]. Furthermore, self-compassion is defined as the process of noticing suffering, where this awareness could be in oneself or others. According to Neff [16], three components are considered important for self-compassion: kindness and understanding towards oneself, understanding that suffering is part of life and being in the present (mindfulness). Acting self-compassionately towards oneself allows an individual to be compassionate to others, as being self-compassionate allows an understanding that failure and suffering are part of life and we all share “human fallibility” [16,17]. Researchers found that this multidimensional construct can decrease mental health difficulties and enhance wellbeing [18,19]. Self-compassion was found to have a strong association with improving wellbeing [20,21] and according to Neff [22], the research appears to be robust. For instance, a meta-analysis in 2015 found from a sample of 16,416 participants that individuals with higher self-compassion had greater wellbeing [23]. Research found that wellbeing is important for health and happiness [24], and therefore, increasing wellbeing can greatly benefit the mental and physical health of an individual [25].

Self-compassion is a skill that can be learned and practised, as it is not a fixed personality trait [22]. Self-compassion and interventions research has grown considerably over the last 20 years, and so far over 4000 journal articles and dissertations have been published [22]. Self-compassion interventions were found to significantly increase wellbeing [16,19,26,27]. Additionally, self-compassion interventions were found to be effective for individuals that experienced trauma, as shown by Winders et al.’s [28] systematic review. The review found 35 studies that increased self-compassion for individuals with PTSD in community and clinical populations, where the findings suggested that the participants experienced reduced symptomatology and decreased impact of trauma exposure [28]. These findings were echoed by a meta-analysis in 2021, which found 12 studies that investigated self-compassion-focused therapy among individuals with posttraumatic stress disorder. Concluding that longer interventions were more effective and overall that the self-compassion interventions provided a protected effect [29]. Self-compassion interventions have been used to target a range of different populations and found to be effective at reducing trauma [28], such as trauma recovery for refugees and asylum-seekers [30]. Ultimately, the interventions work towards reducing the effects of trauma and improving wellbeing and mental health [31,32].

The existing literature recognises the critical role played by self-compassion towards increased wellbeing in healthcare workers [33,34,35]. Self-report data from Kemper et al. [36] highlighted a strong correlation between self-compassion and physical health, mental health, and perceived stress in trainee healthcare workers and clinicians. More recently, Steen et al. [37] reviewed two decades of research regarding the influence of self-compassion and concluded this to be a protective factor and buffer against poor mental health and in maintaining wellbeing among midwives and nurses. Self-compassion was also shown to be significantly and negatively associated with burnout in nurses [38,39,40], and similarly, Prudenzi et al. [41] also reported self-compassion to significantly predict burnout in NHS staff, but interestingly not psychological distress or fatigue. Further, online compassion-focused meditation was noted to significantly improve confidence and compassionate care in healthcare professionals [42]. Self-compassion as an intervention approach was shown to be primitive yet promising [43]. For instance, Neff et al. [44] investigated the efficacy of six one-hour sessions focusing on self-compassion, including practices such as compassionate self-talk, in a population of healthcare professionals. According to the authors, self-compassion was cited to be the primary mechanism of program effectiveness, resulting in enhanced wellbeing and decreased stress. Similarly, Franco and Christie [45] found advantageous outcomes following a one-day self-compassion training programme, such as increased resilience and decreased burnout, anxiety and stress in a sample of nurses. Both studies [45,46] also investigated STS as a further outcome measure; however, at present, there remains a lack of literature exploring the efficacy of self-compassion interventions for healthcare workers, particularly in recognition of its potential for reducing STS. Understanding the link between STS and self-compassion may provide new insight into real-world application and encouragement for its importance in individual intervention and compassionate cultures.

We aimed to synthesise data across studies to provide insight into the role of self-compassion in STS to inform training and practice. Our research question was as follows: Are self-compassion interventions efficacious in reducing secondary traumatic stress in healthcare worker populations?

## 2. Materials and Methods

### 2.1. Protocol Registration

This systematic review was conducted and reported in accordance with the Preferred Reporting Items for Systematic Reviews and Meta-Analyses (PRISMA) statement [46]. The review was registered with The International Prospective Register of Systematic Reviews (PROSPERO; registration number CRD42022359192) in September 2022 and updated in October 2022.

### 2.2. Search Strategy

Literature searches were conducted using the ProQuest, PsycINFO, ScienceDirect, Google Scholar, and EBSCO databases, and these were searched by two independent reviewers (A.R. and M.D.) using a combination of keywords: “Health personnel,” “Health Care Staff,” “Secondary Traumatic Stress,” and “Self-Compassion”. These search terms were determined with the assistance of a subject librarian. There were no date restrictions on searches, although searches were limited to English-language papers. In addition, expert consultation was sought from one academic in the field to assess whether any studies were missed (F.N.). Searches were conducted between October 2022 and February 2023.

### 2.3. Eligibility Criteria

All studies included examined the effect of self-compassion-based interventions (including predominantly compassion elements) on healthcare professionals (including nurses, physicians, and healthcare assistants, with no restriction on the sex, age, and ethnicity of the participants). Studies that did not measure secondary trauma (assessed using validated tools, such as the Professional Quality of Life scale [47]) were excluded. There were no restrictions on the publication status. Table 1 presents our inclusion and exclusion criteria. 

### 2.4. Data Extraction

In the early stage of the review, two reviewers (A.R. and M.D.) screened abstracts for eligibility to categorise studies as either not relevant or potentially relevant. Full texts were then assessed for potentially relevant studies. Studies that were considered ambiguous with respect to the inclusion criteria were discussed, and a consensus was reached for all articles included. The following data was then extracted from each paper by A.R. and agreed upon and verified by M.D.: author names and date, sample and setting, intervention details, measures, and findings [49] (see Table 2).

### 2.5. Quality Assessment of the Included Studies: Assessing the Risk of Bias

The Newcastle–Ottawa Scale [54] was used to appraise the methodological quality and risk of bias for non-randomised studies (see Table 3 and Table 4). Two authors conducted the quality scoring for each study (A.R. and M.D.) using marks from 0–5 for the risk of bias for cohort studies (high risk: 1, medium risk: 2–3, low risk: 4–5), and 0–9 for the risk of bias for case–control study (high risk: 0–3, medium risk: 4–6, low risk: 7–9) [55]. The cohort studies’ top score was reduced from 9 to 5 due to the removal of the subsections; “Selection of non-exposed cohort”, “Demonstrate outcome assessed before intervention” and “Comparability of cohorts on basis of design (*) or analysis (*)”. This was due to these studies not having an intervention group, and all studies targeted STS, which was present at the start. Because the NOS originated in medical research, the word “exposure” was adjusted to “intervention” (e.g., “Ascertainment of intervention”) to be more appropriate for the context of the research.

### 2.6. Quality Assessment of the Review

The appraisal of the methodological quality for this systematic review was based on AMSTAR-2 (a measurement tool designed to assess the quality of systematic reviews [56]). Two independent raters assessed the quality of this review (A.R. and M.D.). It was confirmed that the research question for the review specified a population, intervention, and outcome. The review contained an explicit statement that the review methods, search strategy and inclusion and exclusion criteria were established prior to the conduct of the review and registered with PROSPERO. The review used a comprehensive literature search, the authors performed study selection and date extraction in duplicate (A.R. and M.D.), and the included studies were described in adequate detail. The risk of bias for the included studies was assessed using the Newcastle–Ottawa scale and the results of this informed the discussion.

### 2.7. Analysis

A meta-analysis was considered unsuitable due to there being insufficient studies and study design. The findings are summarised using a systematic narrative approach.

**Table 3:** Assessment of the quality of the studies (non-randomised trials cohort; five studies; * indicates this is present).

Assessment of Risk of Bias for Cohort Studies (The Newcastle–Ottawa Scale)									
		Selection		Comparability		Outcome		Number of Stars (0–5)	
References	Representativeness of Exposed Cohort	Selection of Non-Exposed Cohort	Ascertainment of Intervention	Demonstrate Outcome Assessed before Intervention	Comparability of Cohorts on Basis of Design (*) or Analysis	Assessment of Outcome	Follow-Up Long Enough	Adequacy of Follow-Up	
Dewidar et al. (2022) [50]	*	N/A	*	N/A	N/A	*	*		4
McVicar et al. (2020) [51]		N/A	*	N/A	N/A	*			2
Neff et al. (2020) [44]		N/A	*	N/A	N/A	*			2
Marconi et al. (2019) [52]		N/A	*	N/A	N/A	*			2
Delaney (2018) [53]		N/A	*	N/A	N/A	*			2

**Table 4:** Assessment of the quality of the studies (non-randomised trials case–control; one study; * indicates this is present).

	Assessment of Risk of Bias for Case Control Studies (The Newcastle-Ottawa Scale)								
	Selection			Comparability		Outcome		Number of Stars (0–9)	
References	Is the Case Definition Adequate?	Representativeness of the Case	Selection of Controls	Definition of Controls	Comparability of Cases and Controls on the Basis of Design (*) or Analysis (*)	Ascertainment of Intervention	Same Method of Ascertainment for Case and Controls	Non-Response Rate	
Franco and Christie (2021) [45]	*	*	*		*	*	*		6

## 3. Results

### 3.1. Search Results

**Figure 1:**
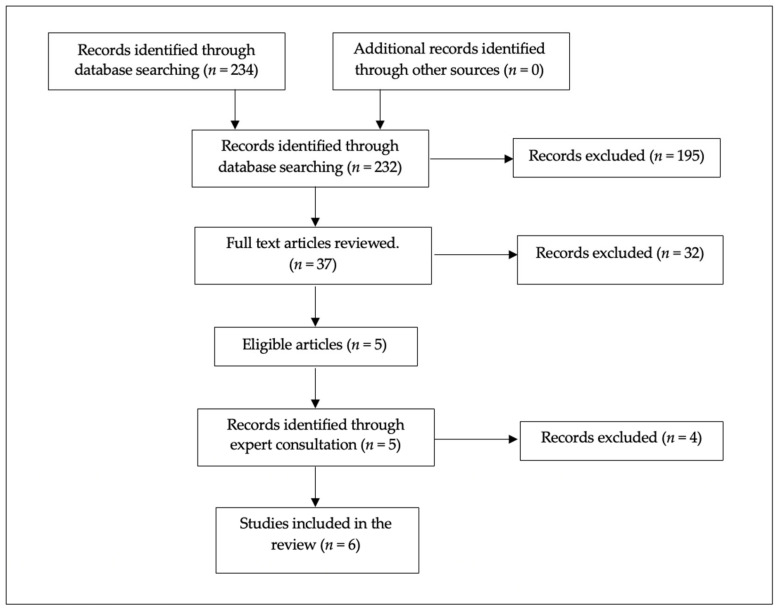
PRISMA flow diagram of the article selection process.

The selection of studies is shown in Figure 1 using the PRISMA flow diagram. The search identified 234 articles that were screened for eligibility. Duplicates and corrigenda were removed (105) and the remaining articles that contained both a secondary traumatic stress and compassion component and healthcare in the title and abstract were considered. Thirty-seven full-text articles were read in full by two reviewers (A.R. and M.D.). Finally, studies that did not contain self-compassion as a main component of the intervention were removed, as were studies that did not report STS specifically. A total of six studies were considered eligible for the final analysis. Expert consultation was then sought (F.N.), which offered an additional five papers for consideration. On inspection of the full texts by two reviewers (A.R. and M.D.), one additional study was considered eligible for the final analysis; therefore, six studies were included in the final analysis.

### 3.2. Characteristics of the Included Studies

#### 3.2.1. Published Year

All studies were published in the last 7 years (2018–2022).

#### 3.2.2. Location of Research

Two studies were conducted in the UK [51,53], two in the USA [44,45], one in Italy [52], and one in Egypt [50].

#### 3.2.3. Design

Four studies used a single-group pre–post test design [44,51,52,53], one study used a single-group pre–post test and follow-up design [50], and one study used a comparison group between-subject pre–post test and follow-up design [45].

#### 3.2.4. Participant Demographics

All studies (that reported gender balance) recruited more females than males, including one study that recruited women only [53], and the age ranges across all six studies varied. The sample size ranged from 13 [53] to 50 participants [50], totaling 194 participants overall.

#### 3.2.5. Occupational Field

Participants in three studies worked as nurses [45,50,53]; one study involved health visitors only [51]; and two studies were a mix of healthcare staff, such as nurses, physicians, and social workers [44,52].

#### 3.2.6. Scale

All studies [44,45,50,51,52,53] used the Professional Quality of Life Scale (ProQOL [47]) to measure STS.

#### 3.2.7. Intervention Design and Treatment Effectiveness

One study utilised a mindful self-compassion intervention containing mindfulness meditation, loving kindness meditation, compassion meditation, and the core principles of compassion and self-compassion [53]. One study used a compassionate-mind-model-based curriculum for health visitor training students centering on compassion and self-compassion training [51]. Two studies used the Self-Compassion for Healthcare Communities (SCHC) program, which is an adaptation of the Mindful Self-Compassion course developed by Neff and Germer [57] but specifically targeted at healthcare communities. The SCHC program has a focus on self-compassion training, with one study using the full programme [44] and one using a 6h adaption of the SCHC programme [45]. One study used a compassion-orientated mindfulness programme (COMP), which is a compassion and mindfulness-based training programme that includes self-compassion and empathy training [52], and the final study used a compassion training programme containing training on concepts of compassion and self-compassion, professional quality of life, compassionate responses and empathy [50].

Four of the six studies reported a significant reduction in STS following the intervention [44,50,51,53]. Of these, two reported effect sizes of 0.67 [44] and 0.82 [53], indicating a large effect. Two did not report an effect size [50,51]. Two studies reported a reduction in STS, but this was non-significant [45,52].

#### 3.2.8. Quality Scoring: Assessing the Risk of Bias

Four studies were towards the lower limit of medium risk [44,51,52,53], one study was towards the upper limit of medium risk [45], and one study was at the lower limit of low risk [50]. Representativeness of the cohort and follow-up assessments were common weaknesses.

## 4. Discussion

This PRISMA-based systematic review appraised the quality and quantity of evidence for eligible studies evaluating the effects of self-compassion training regarding secondary traumatic stress (STS) in the healthcare population. Six intervention studies comprising 194 participants met all the eligibility criteria for in-depth review and assessment. STS was seen to reduce in all studies, although, in two of these, this reduction was non-significant [45,52]. Improvements were also reported across other outcomes, such as anxiety, depression, self-compassion, compassion fatigue, and burnout. The rigour of the included studies was medium. Thus, findings from this systematic review indicate that self-compassion-based interventions may have applications for improving secondary traumatic stress in healthcare populations, although the low study quantity found indicates a gap in the research in this area.

The total length of the interventions varied from one day to 12 months; however, in the one high-quality study with significant results [50], eight one-hour sessions over eight weeks were employed. This was also seen to be maintained at the three-month follow-up. Another study showed significant improvements within eight weeks [53], although this study did not include a follow-up, making it difficult to ascertain whether the results were maintained. Of the other two studies with significant results, the intervention duration ranged from four weeks [44] to 52 weeks [51]. Although both studies saw significant results, as neither included a follow-up assessment, it is difficult to know whether either the 52-week or 4-week programme is adequate to maintain results. Thus, based on the six studies included in this systematic review, the most robust preliminary evidence exists for self-compassion interventions of at least an eight-week duration, spread across weekly sessions. It is worth noting, however, that none of these studies were RCTs, and therefore, more high-quality research is needed to draw conclusions about the most effective length of intervention.

The majority of studies included in this review were set in Western countries, with the exception of Dewidar et al. [50]. It is worth noting that self-compassion can be experienced differently in different cultures. More individualistic cultures may be more success-driven, and thus, more competitive and self-critical, in turn, making self-compassion more challenging [58,59,60]. Self-compassion and the benefits associated with it may not be as easy to increase in cultures where it is already naturally higher. Dewidar et al.’s study [50] from Egypt reported a significant reduction in STS, which implies positive preliminary evidence that self-compassion training may be effective in non-Western cultures, although further research is required to build on this.

The studies had a small, majority White female sample, with a mean age typically over 30, recruited mainly from one hospital in their respective geographical areas, and were without a control group (with the exception of Franco and Christie [45]). While this limits the generalisability of the findings, implications on the population not represented in aforementioned studies should be considered also. Given the high turnover of healthcare staff, particularly in psychiatric settings and low-paid roles, which are typically taken on by younger generations, consideration should be given to the impact of STS when joining the healthcare workforce in the teen years and early twenties. Organisations that employ the use of self-compassion interventions in their initial training may therefore see increased retainment of their staff; however, additional research is required to understand the development of STS and the impact of self-compassion interventions on new younger staff [61,62]. That being said, McVicar et al. [51] found that “years in practice” was strongly positively correlated with STS, adhering to the idea of STS accumulating over time. Thus, while this finding is significant, self-compassion interventions may provide a preventative, rather than reactive, approach to STS if introduced earlier on.

It is worth also noting that healthcare workers often have time-limited schedules, and further training demands may incur unintentional stress [63]. As the outcomes for STS were non-significant, Franco and Christie’s [45] time-intensive intervention does not support the notion that this would be effective. Due to the study’s ceiling effect for resiliency activation and lack of power, additional research should endeavor to determine organisational feasibility and whether interventions over a shorter period remain efficacious. Further, while requesting participants to practice learned techniques after sessions proved advantageous, this intensifies the time commitment, potentially overwhelming staff [64]. These considerations should be noted when assessing the acceptability of self-compassion interventions for this population. However, it is an organisation’s responsibility to ensure that its healthcare workers are protected and valued, further promoting attentiveness and compassion, and thus, a higher standard of practice. Systemic change is required in order to improve working conditions. However, providing timely support for healthcare staff to develop coping skills may assist with mediating and moderating distress. This may lead to downstream effects, such as enhanced outcomes for both patients and healthcare workers. Healthcare populations may feel more supported, less alone, or less likely to use maladaptive strategies to cope with the difficulties faced in their daily working lives. Similarly, patients may note greater compassion, attentiveness, and a higher overall standard of care, contributing to sustained outcomes for both.

Overall, the current collection of evidence regarding the efficacy of self-compassion interventions for STS in a healthcare worker population is both mixed and promising. The quality of the studies included was medium overall: four studies were assessed to bear a medium risk of bias [44,51,52,53], and two studies were assessed to bear a low risk of bias [45,50]. Of the non-controlled studies, most did not address “representativeness of the exposed cohort” and “follow-up adequacy”. Future research may benefit from recruiting a more ethnically diverse group of workers and a more even distribution of gender and age. Additionally, future research may benefit from including a follow-up assessment to ascertain whether positive results were maintained. Overall, more methodologically robust controlled studies of self-compassion interventions for STS in health worker populations are required to address these issues. The one study assessed that did provide a control group and had a low risk of bias had non-significant results [45]. Although the majority of studies did show a significant reduction in STS [44,50,51,53], none of these contained a control group, making it difficult to ascertain whether the intervention was successful as a result of the content, or just the awareness gained from discussing and being educated about STS. Future research may benefit from including an active control to determine the specific impact of self-compassion training on STS. Although future research is required to address the above concerns, and there is a recognised need to address the growing concern around STS in this population [8,14], future research may also benefit from understanding the environmental factors that could give rise to STS in this population. A combination of assessing treatment options for STS, in addition to understanding what may prevent STS from occurring in the first place, would benefit the healthcare worker population.

While this systematic review offers helpful insights into the application of self-compassion for STS in a healthcare worker context, several limitations should be noted. This review comprised a total of six studies, with most studies being the first of their kind or pilot studies. Further, study homogeneity was largely absent, as studies varied in terms of the sample, geographical location, time-length of the intervention, and time-intensity of the intervention. Additionally, the majority of participants across the selected studies were self-selected [65], and therefore, may be more likely to show an interest in learning skills or to continue practicing skills following the termination of the intervention. This may bias findings post-intervention and at follow-up compared with individuals that are required to take part in the intervention as compulsory training and may incur higher attrition rates or criticisms of the intervention. Furthermore, the majority of studies did not use a control group, making it difficult to ascertain whether the results were due to self-compassion or just the consideration of STS. Additionally, standardisation of the self-compassion programmes has not been done. Therefore, each study may have offered very different interventions. Further to this, it is not possible to know which components of these programmes were effective, and without this knowledge, a future programme cannot be developed with solid evidence. Lastly, it is important to consider that the authors are healthcare and psychology specialists. Thus, this systematic review may be subject to researcher bias through bias interpretation, although each author was mindful of this throughout. Service users or family members may have different insights about HCWs’ wellbeing, and this has not been evaluated. Despite these considerations, this review provides an initial look at the potential of self-compassion interventions to reduce STS in healthcare populations. Further research should build upon these foundations to substantiate the credibility of the findings and conclusions drawn. Furthermore, studies that were not yet published or studies that were published in languages other than English were not considered, and thus, additional evidence for the efficacy of self-compassion interventions for STS may have been overlooked. Additionally, this review excluded studies where self-compassion was not the main component of the intervention, meaning self-compassion may have been present in other excluded studies but not explicitly stated, and if so, these also may have been overlooked. Therefore, future reviews may benefit from using a boarder definition of self-compassion.

## 5. Conclusions

The six studies included in this review indicate preliminary evidence that self-compassion training may be beneficial for improving STS in healthcare worker populations. However, there is a gap in the research regarding this, where the evidence base for this area is under-developed and has largely focused on a predominantly female population in Western countries. More male healthcare workers and both male and female workers from non-Western countries need to be recruited. The overall research quality of these six studies was medium, often lacking the representativeness of cohort and follow-up assessments. Future research about STS in healthcare workers should improve the recruitment and assessment strategies, as well as utilise control groups. The findings will help healthcare managers and employers identify staff wellbeing approaches focused on STS.

## Figures and Tables

**Table 1:** Extended PICO for this systematic review [48].

Review Question	How Effective is Self-Compassion in Treating Secondary Traumatic Stress in a Health Care Worker Population?	
	Inclusion Criteria	Exclusion Criteria
Population	Healthcare workers > 18 years old	<18 years old and workers not in healthcare roles
Intervention	Interventions that focused on self-compassion ^1^	Other interventions: resilience interventions, stress reduction interventions, and mindfulness interventions
Comparator	Any comparator including non-intervention	
Outcomes	Secondary traumatic stress	Other outcomes
Study design	Empirical intervention studies	Single-participant case studies, cross-sectional studies, qualitative studies, reviews, discussion articles, and theoretical articles
Other	Published in a peer-reviewed journal in the English language	

^1^ Training that did not focus on self-compassion was excluded (e.g., resilience training), training that only had a mindfulness element with no self-compassion was excluded.

**Table 2:** Study details of selected papers exploring the effects of self-compassion training on secondary traumatic stress in healthcare workers.

Author(s) (Year)	Sample and Setting	Intervention Details	Measures	Findings
Dewidar et al. (2022) [50]	50 mental health nurses at the Psychiatry Centre of Tanta University in Egypt. 13 male and 37 female with a mean age of 28 (21–40). Single-group pre–post 3-month follow-up design.	8 × 1 h weekly sessions delivered over 8 weeks with a focus on compassion, self-compassion, compassionate responding, empathy, and self-awareness.	Professional Quality of Life Scale.	Significant reduction in secondary traumatic stress, with a decrease of 17.1%.
McVicar et al. (2020) [51]	26 practitioners on an NHS health visitor course in the east of England. Single-group, within-subject, pre–post design.	52 sessions using a Compassionate-Mind-Model-based curriculum delivered weekly over 52 weeks.	Professional Quality of Life Scale.	Significant reduction in secondary traumatic stress with a decrease of 16.5% between the pre- and post-test and a small-to-medium effect size (0.20–0.42).
Neff et al. (2020) [44]	23 healthcare workers from a hospital in the Southwestern United States (35% in ancillary services, 22% physicians, 17% social workers, 13% in therapeutic services, 9% nurses, and 4% other). Sample was 96% female with a mean age = 37.57 (27–60) and 74% White, 17% Latino, 4% Asian American, and 4% other. Single-group, within-subject, pre–post design.	Self-compassion for healthcare communities (SCHC) programme, which is an adaption of the Mindful Self-Compassion programme. 4 × 1.5 h sessions held over 4 weeks.	Professional Quality of Life Scale.	Significant reduction in secondary traumatic stress with a decrease of 26% and a large effect size (0.67).
Marconi et al. (2019) [52]	34 psychiatric health professionals with a mean age of 50 in a hospital in Italy. Single-group, within-subject, pre–post test design.	Compassion-orientated mindfulness programme. 3 h classes fortnightly over 18 weeks.	Professional Quality of Life Scale.	Reduction in secondary traumatic stress but the results were non-significant.
Delaney (2018) [53]	13 female nurses across departments of cancer care, cardiology, maternity, midwifery, intensive care, and urology in a UK hospital. Mean age of 44. Single-group, within-subject, pre–post test design.	Mindful Self-Compassion training. 8 × 2.5 h sessions weekly plus a half-day retreat.	Professional Quality of Life Scale.	Significant reduction in secondary traumatic stress with a decrease of 15% and a large effect size (0.82).
Franco and Christie (2021) [45]	48 pediatric nurses at a pediatric hospital in Texas. A waitlist control between subjects with a pre–post follow-up design. The intervention group had 22 participants with 21 females and a mean age of 46. The control group had 26 participants with 22 females and a mean age of 38.	A short 6 h adaption of the Self-Compassion for Healthcare Communities programme. 6 × 1 h sessions delivered throughout one day.	Professional Quality of Life Scale.	Reduction in secondary traumatic stress in the intervention group but the results were non-significant.

## Data Availability

No new data were created.

## References

[1] Figley C.R. (1995). Compassion fatigue as secondary traumatic stress disorder: An overview. Compassion Fatigue: Coping with Secondary Traumatic Stress Disorder in Those Who Treat the Traumatized.

[2] Sinclair S., Raffin-Bouchal S., Venturato L., Mijovic-Kondejewski J., Smith-MacDonald L. (2017). Compassion fatigue: A meta-narrative review of the healthcare literature. Int. J. Nurs. Stud..

[3] Ludick M., Figley C.R. (2017). Toward a mechanism for secondary trauma induction and reduction: Reimagining a theory of secondary traumatic stress. Traumatology.

[4] Kotera Y., Young H., Maybury S., Aledeh M. (2022). Mediation of Self-Compassion on Pathways from Stress to Psychopathologies among Japanese Workers. Int. J. Environ. Res. Public Health.

[5] Kotera Y., Dosedlova J., Andrzejewski D., Kaluzeviciute G., Sakai M. (2021). From Stress to Psychopathology: Relationship with Self-Reassurance and Self-Criticism in Czech University Students. Int. J. Ment. Health Addict..

[6] Kotera Y., Ozaki A., Miyatake H., Tsunetoshi C., Nishikawa Y., Kosaka M., Tanimoto T. (2022). Qualitative Investigation into the Mental Health of Healthcare Workers in Japan during the COVID-19 Pandemic. Int. J. Environ. Res. Public Health.

[7] Figley C.R., Figley C.R., McCubbin H.I. (1983). Catastrophes: An overview of family reactions. Stress and the Family, Vol. II: Coping with Catastrophe.

[8] Beck C.T. (2011). Secondary traumatic stress in nurses: A systematic review. Arch. Psychiatr. Nurs..

[9] Orrù G., Marzetti F., Conversano C., Vagheggini G., Miccoli M., Ciacchini R., Panait E., Gemignani A. (2021). Secondary Traumatic Stress and Burnout in Healthcare Workers during COVID-19 Outbreak. Int. J. Env. Res. Public Health.

[10] Morrison L.E., Joy J.P. (2016). Secondary traumatic stress in the emergency department. J. Adv. Nurs..

[11] Duffy E., Avalos G., Dowling M. (2015). Secondary traumatic stress among emergency nurses: A cross-sectional study. Int. Emerg. Nurs..

[12] Ratrout H.F., Hamdan-Mansour A.M. (2020). Secondary traumatic stress among emergency nurses: Prevalence, predictors, and consequences. Int. J. Nurs. Pract..

[13] Roden-Foreman J.W., Bennett M.M., Rainey E.E., Garrett J.S., Powers M.B., Warren A.M. (2017). Secondary traumatic stress in emergency medicine clinicians. Cogn. Behav. Ther..

[14] Nimmo A., Huggard P. (2013). A systematic review of the measurement of compassion fatigue, vicarious trauma, and secondary traumatic stress in physicians. Australas. J. Disaster Trauma Stud..

[15] Greinacher A., Derezza-Greeven C., Herzog W., Nikendei C. (2019). Secondary traumatization in first responders: A systematic review. Eur. J. Psychotraumatol..

[16] Neff K. (2003). Self-compassion: An alternative conceptualization of a healthy attitude toward oneself. Self Identity.

[17] Kotera Y., Llewellyn-Beardsley J., Charles A., Slade M. (2022). Common Humanity as an Under-acknowledged Mechanism for Mental Health Peer Support. Int. J. Ment. Health Addict..

[18] Finlay-Jones A.L. (2017). The relevance of self-compassion as an intervention target in mood and anxiety disorders: A narrative review based on an emotion regulation framework. Clin. Psychol..

[19] MacBeth A., Gumley A. (2012). Exploring compassion: A meta-analysis of the association between self-compassion and psychopathology. Clin. Psychol. Rev..

[20] Neff K.D., Long P., Knox M.C., Davidson O., Kuchar A., Costigan A., Williamson Z., Rohleder N., Tóth-Király I., Breines J.G. (2018). The forest and the trees: Examining the association of self-compassion and its positive and negative components with psychological functioning. Self Identity.

[21] Phillips W.J., Hine D.W. (2021). Self-compassion, physical health, and health behaviour: A meta-analysis. Health Psychol. Rev..

[22] Neff K.D. (2023). Self-Compassion: Theory, Method, Research, and Intervention. Annu. Rev. Psychol..

[23] Zessin U., Dickhäuser O., Garbade S. (2015). The Relationship Between Self-Compassion and Well-Being: A Meta-Analysis. Appl. Psychol. Health Well-Being.

[24] Seligman M.E.P. (2011). Flourish: A Visionary New Understanding of Happiness and Well-Being.

[25] Hernandez R., Bassett S.M., Boughton S.W., Schuette S.A., Shiu E.W., Moskowitz J.T. (2018). Psychological well-being and physical health: Associations, mechanisms, and future directions. Emot. Rev..

[26] Warren R., Smeets E., Neff K. (2016). Self-criticism and self-compassion: Risk and resilience: Being compassionate to oneself is associated with emotional resilience and psychological well-being. Curr. Psychiatry.

[27] Kotera Y., Cockerill V., Chircop J., Kaluzeviciute G., Dyson S. (2021). Predicting Self-Compassion in UK Nursing Students: Relationships with Resilience, Engagement, Motivation, and Mental Wellbeing. Nurse Educ. Pract..

[28] Winders S.J., Murphy O., Looney K., O’Reilly G. (2020). Self-compassion, trauma, and posttraumatic stress disorder: A systematic review. Clin. Psychol. Psychother..

[29] Luo X., Che X., Lei Y., Li H. (2021). Investigating the Influence of Self-Compassion-Focused Interventions on Posttraumatic Stress: A Systematic Review and Meta-Analysis. Mindfulness.

[30] Aizik-Reebs A., Amir I., Yuval K., Hadash Y., Bernstein A. (2022). Candidate mechanisms of action of mindfulness-based trauma recovery for refugees (MBTR-R): Self-compassion and self-criticism. J. Consult. Clin. Psychol..

[31] Fancourt D., Finn S. (2019). What Is the Evidence on the Role of the Arts in Improving Health and Well-Being? A Scoping Review.

[32] Schlesselman L.S., Cain J., DiVall M. (2020). Improving and Restoring the Well-being and Resilience of Pharmacy Students during a Pandemic. Am. J. Pharm. Educ..

[33] Kotera Y. (2021). De-stigmatising self-care: Impact of self-care webinar during COVID-19. Int. J. Spa Wellness.

[34] Kotera Y., Ozaki A., Miyatake H., Tsunetoshi C., Nishikawa Y., Tanimoto T. (2021). Mental health of medical workers in Japan during COVID-19: Relationships with loneliness, hope and self-compassion. Curr. Psychol..

[35] Kotera Y., Liu G., Colman R., Young H., Ozaki A., Miyatake H., Kosaka M., Tanimoto T. (2023). A longitudinal study of mental health in healthcare workers in Japan during the initial phase of COVID-19 pandemic: Comparison with the general population. Curr. Psychol..

[36] Kemper K.J., Mo X., Khayat R. (2015). Are mindfulness and self-compassion associated with sleep and resilience in health professionals?. J. Altern. Complement. Med..

[37] Steen M., Javanmard M., Vernon R. (2021). The influence of self-compassion upon midwives and nurses: A scoping review: Self-compassion and midwives and nurses. Evid. Based Midwifery.

[38] Durkin M., Beaumont E., Hollins Martin C.J., Carson J. (2016). A pilot study exploring the relationship between self-compassion, self-judgement, self-kindness, compassion, professional quality of life and wellbeing among UK community nurses. Nurse Educ. Today.

[39] Ruiz-Fernández M.D., Ramos-Pichardo J.D., Ibáñez-Masero O., Carmona-Rega M.I., Sánchez-Ruiz M.J., Ortega-Galán M. (2021). Professional quality of life, self-compassion, resilience, and empathy in healthcare professionals during COVID-19 crisis in Spain. Res. Nurs. Health.

[40] Kotera Y., Maxwell-Jones R., Edwards A.M., Knutton N. (2021). Burnout in professional psychotherapists: Relationships with self-compassion, work–life balance, and telepressure. Int. J. Environ. Res. Public Health.

[41] Prudenzi A., Graham C.D., Flaxman P.E., O’Connor D.B. (2022). Wellbeing, burnout, and safe practice among healthcare professionals: Predictive influences of mindfulness, values, and self-compassion. Psychol. Health Med..

[42] Rao N., Kemper K.J. (2017). Online Training in Specific Meditation Practices Improves Gratitude, Well-Being, Self-Compassion, and Confidence in Providing Compassionate Care Among Health Professionals. J. Evid.-Based Complement. Altern. Med..

[43] Kotera Y., Van Gordon W. (2021). Effects of Self-Compassion Training on Work-Related Well-Being: A Systematic Review. Front. Psychol..

[44] Neff K.D., Knox M.C., Long P., Gregory K. (2020). Caring for others without losing yourself: An adaptation of the Mindful Self-Compassion Program for Healthcare Communities. J. Clin. Psychol..

[45] Franco P.L., Christie L.M. (2021). Effectiveness of a One Day Self-Compassion Training for Pediatric Nurses’ Resilience. J. Pediatr. Nurs..

[46] Page M.J., McKenzie J.E., Bossuyt P.M., Boutron I., Hoffmann T.C., Mulrow C.D., Shamseer L., Tetzlaff J.M., Akl E.A., Brennan S.E. (2021). The PRISMA 2020 statement: An updated guideline for reporting systematic reviews. BMJ.

[47] Stamm B.H. Professional Quality of Life: Compassion Satisfaction and Fatigue Version 5 (ProQOL). www.proqol.org.

[48] Kotera Y., Sheffield D., Van Gordon W. (2019). The applications of neuro-linguistic programming in organizational settings: A systematic review of psychological outcomes. Hum. Resour. Dev. Q..

[49] Kotera Y., Lyons M., Vione K.C., Norton B. (2021). Effect of Nature Walks on Depression and Anxiety: A Systematic Review. Sustainability.

[50] Dewidar A.E.-S.M., Gado E.M., Gemeay E.M., Sabra A.I. (2022). Effect of Training Program about Compassion on Professional Quality of Life of Mental Health Nurses. Int. Egypt. J. Nurs. Sci. Res..

[51] McVicar A., Pettit A., Knight-Davidson P., Shaw-Flach A. (2021). Promotion of professional quality of life through reducing fears of compassion and compassion fatigue: Application of the Compassionate Mind Model to Specialist Community Public Health Nurses (Health Visiting) training. J. Clin. Nurs..

[52] Marconi A., Balzola M.A., Gatto R., Soresini A., Mabilia D., Poletti S. (2019). Compassion-oriented mindfulness-based program and health professionals: A single-centered pilot study on burnout. Eur. J. Ment. Health.

[53] Delaney M.C. (2018). Caring for the caregivers: Evaluation of the effect of an eight-week pilot mindful self-compassion (MSC) training program on nurses’ compassion fatigue and resilience. PLoS ONE.

[54] Wells G., Shea B., O’Connell D., Peterson J., Welch V., Losos M., Tugwell P. (2000). The Newcastle-Ottawa Scale (NOS) for Assessing the Quality of Nonrandomised Studies in Meta-Analyses. http://www.evidencebasedpublichealth.de/download/Newcastle_Ottowa_Scale_Pope_Bruce.pdf.

[55] Kotera Y., Correa Vione K. (2020). Psychological Impacts of the New Ways of Working (NWW): A Systematic Review. Int. J. Environ. Res. Public Health.

[56] Shea B.J., Reeves B.C., Wells G., Thuku M., Hamel C., Moran J., Moher D., Tugwell P., Welch V., Kristjansson E. (2017). AMSTAR 2: A critical appraisal tool for systematic reviews that include randomised or non-randomised studies of healthcare interventions, or both. BMJ.

[57] Neff K.D., Germer C.K. (2013). A Pilot Study and Randomized Controlled Trial of the Mindful Self-Compassion Program. J. Clin. Psychol..

[58] Montero-Marin J., Kuyken W., Crane C., Gu J., Baer R., Al-Awamleh A.A., Akutsu S., Araya-Véliz C., Ghorbani N., Chen Z.J. (2018). Self-Compassion and Cultural Values: A Cross-Cultural Study of Self-Compassion Using a Multitrait-Multimethod (MTMM) Analytical Procedure. Front. Psychol..

[59] Kotera Y., Van Laethem M., Ohshima R. (2020). Cross-cultural comparison of mental health between Japanese and Dutch workers: Relationships with mental health shame, self-compassion, work engagement and motivation. Cross Cult. Strateg. Manag..

[60] Kotera Y., Tsuda-McCaie F., Maughan G., Green P. (2021). Cross-cultural comparison of mental health in social work students between UK and Ireland: Mental health shame and self-compassion. Br. J. Soc. Work..

[61] Kotera Y., Adhikari P., Sheffield D. (2020). Mental health of UK hospitality workers: Shame, self-criticism and self-reassurance. Serv. Ind. J..

[62] Kotera Y., Mayer C.-H., Vanderheiden E. (2021). Cross-Cultural Comparison of Mental Health Between German and South African Employees: Shame, Self-Compassion, Work Engagement, and Work Motivation. Front. Psychol..

[63] Kotera Y., Spink R., Brooks-Ucheaga M., Green P., Rawson R., Rhodes C., Chircop J., Williams A., Okere U., Lyte G. (2021). Teaching healthcare professional students in online learning during COVID-19: Reflection of university lecturers. J. Concurr. Disord..

[64] Kotera Y., Sweet M. (2019). Comparative evaluation of neuro-linguistic programming. Br. J. Guid. Couns..

[65] Kotera Y. (2017). A qualitative investigation into the experience of neuro-linguistic programming certification training among Japanese career consultants. Br. J. Guid. Couns..

